# Effect of Punch Surface Microtexture on the Microextrudability of AA6063 Micro Backward Extrusion

**DOI:** 10.3390/mi13112001

**Published:** 2022-11-17

**Authors:** Tatsuya Funazuka, Kuniaki Dohda, Tomomi Shiratori, Syunsuke Horiuchi, Ikumu Watanabe

**Affiliations:** 1 Academic Assembly Faculty of Engineering, University of Toyama, Toyama 930-8555, Japan; 2 Department of Mechanical Engineering, Northwestern University, Evanston, IL 60201, USA; 3 Graduate School of Science and Engineering for Education, University of Toyama, Toyama 930-8555, Japan; 4 Research Center for Structural Materials, National Institute for Materials Science, Tsukuba 205-0047, Japan

**Keywords:** aluminum alloy, microextrusion, microtexture, size effect, tribology

## Abstract

To apply conventional forming processes to microscale processing, the influence of size effects caused by material properties and friction effects must be considered. Herein, the effects of tool surface properties, such as punch surface texture, on microextrusion properties, such as extrusion force, product shape, and product microstructure, were investigated using AA6063 billets as test pieces. Millimeter-scale, microscale, and nanoscale textures were fabricated on the punch surfaces. Punch texturing was conducted by electrical discharge machining or polishing or using a laser process. The extrusion force increased rapidly as the stroke progressed for all punch textures. Comparing the product shapes, the smaller the texture size, the lower the adhesion and the longer the backward extrusion length. The results of material analysis using electron backscatter diffraction show that material flowability is improved, and more strain is uniformly applied when a nanoscale-textured punch is used. By contrast, when a mirror punch was used, material flowability decreased, and strain was applied non-uniformly. Therefore, by changing the surface properties of the punch, the tribology between the tool and material can be controlled, and formability can be improved.

## 1. Introduction

Owing to the rapid miniaturization of various products in recent years, the fabrication of microparts that constitute medical devices has been attracting attention. Thus far, microparts have been manufactured mainly by machining; however, research on microplastic forming has also become active owing to requirements such as reduction in the production cost and flexibility in the product shape [1]. Among such processes, microextrusion processing has attracted considerable industrial attention as a micropart-forming process technology. When extrusion, which is a conventional macroscale machining technology, is applied on a microscale, issues in reproducibility and accuracy arise. Engel et al. clarified the effect of small product dimensions on tribology via microscale double-cup extrusion tests and reported the following findings: as the product dimensions decreased, the tool–billet contact area and the pockets that hold the lubricant decrease and the area of direct contact increases, resulting in increased friction [2]. Additionally, a study on suitable processing temperatures for microforming showed that stable forming is possible at high temperatures, where dislocation migration is activated; moreover, the variation in product accuracy owing to the size effect is reduced [3].

Bunget and Ngaile [4], Xu et al. [5], and Lou et al. [6] reported that ultrasonic vibration contributed to the improvement of the forming load and material flow performance via ultrasonic microextrusion. Chan et al. [7,8] showed that by considering multiple influencing factors, such as material flow pattern, interface condition, and flow stress curves, microforming analysis can be used to accurately predict deformation behavior. In a series of studies on microextrusion [9,10,11,12], Cao et al. examined the effects of microstructure, such as the grain size, shape, and orientation of the billet, and interface conditions, on the processing. They evaluated the plastic deformation behavior of different grain sizes in microextrusion and showed that the larger the grain size, the more easily the extruded part is bent owing to non-uniform deformation and that the difference in grain size affects formability. Furthermore, the effectiveness of the hard coatings in stabilizing friction during processing was investigated. The effectiveness of applying high-strength, low-friction hard coatings to die surfaces was reported, with the deposition of silicon-containing diamond-like carbon (DLC-Si) coating being the most effective.

In microscale forming, the surface becomes rougher relative to the working scale. This surface roughness causes significant changes in the friction and forming behavior [13,14]. Moreover, achieving surface properties suitable for the microscale is difficult, and the optimal tool surface conditions for reducing friction and improving formability should be determined. In the field of microfabrication, microtexturing has been successfully applied to reduce friction and increase the lubrication pocket area on tool surfaces [15,16,17]. Microtexturing is expected to stabilize machinability by reducing the tool contact area and retaining the lubricant.

The effects of microstructure, lubricant, and die coating on forward–backward microextrusion techniques for aluminum alloys have been identified [18,19,20]. Complex material flow in both the forward and backward directions was evaluated experimentally and by simulation; backward extrusion was considerably affected by die and punch friction and microstructure.

These studies revealed that grain size control and tribology at the tool–material interface significantly affect formability parameters, such as forming force and material flow, in microextrusion. The backward extrusion of microscale parts was performed to realize microforming. When the punch surface is provided with an arbitrary surface roughness using abrasive paper, the tool contact area reduces, and material flow improves [21]. However, considerable uncertainty exists regarding the effect of the punch surface texture size on micro backward extrusion processing; therefore, the effect of the texture size from millimeter-scale to nanoscale for optimal tool surface design in microforming should be investigated. In shearing tests, certain examples of nanotextured punches have been applied, and the use of nanotextured punches has resulted in high-precision micro-shearing owing to the reduction of the processing-influenced layer [22,23].

Herein, millimeter- to nanoscale-textured tools were used to stabilize formability by reducing the tool contact area and retaining the lubricant. Microtextures of various scales were applied to the punch surface to investigate the effect of the punch surface properties. The effects of punch surface texture on microextrusion formability were estimated based on the extrusion force, billet shape after forming, amount of adhesion to the punch, and microstructure analysis of the product.

## 2. Materials and Methods

**Figure 1:**
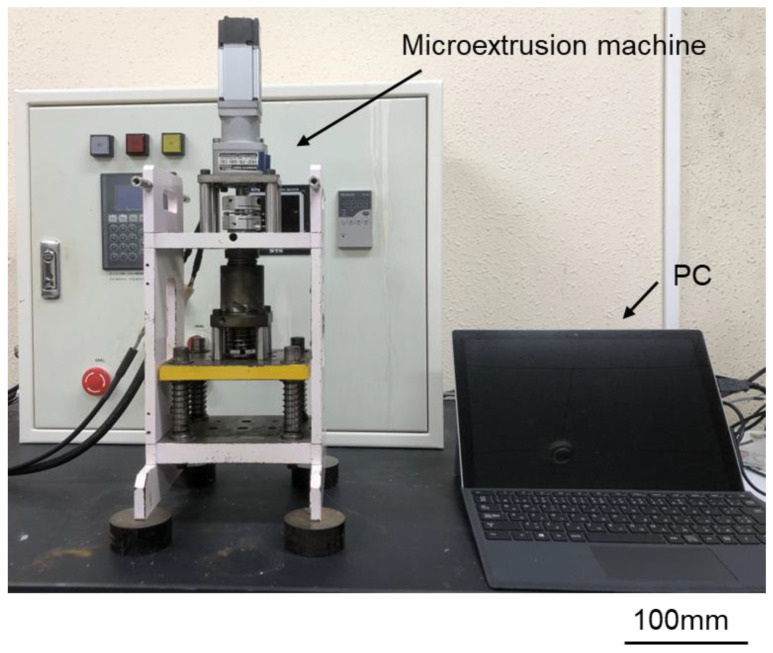
Photograph of the microextrusion machine [21].

The microextrusion machine (Micro Fabrication Laboratory, Japan) used in this study and shown in Figure 1 [21] is a servomotor-driven tabletop screw press that transmits torque directly or is amplified by a servomotor to the screw shaft. The screw axis is connected to the punch via a load cell to control the forming speed and position. The microextrusion machine, die, punch, and other tools as well as the billet have the same dimensions as those in the previous study.

**Figure 2:**
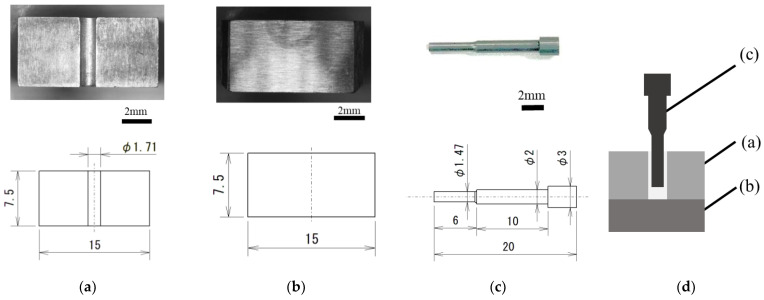
Shape of the die and punch: (**a**) extrusion die, (**b**) jig, and (**c**) punch; (**d**) schematic of backward microextrusion [21].

A schematic of the die and punch used for this study is shown in Figure 2 [21]. The die was divided at the center to remove the billet after extrusion. Moreover, the die had a container inner diameter of φ1.71 mm. The arithmetic mean roughness inside the container was Ra = 0.18 µm. The punch was selected for backward extrusion, and the diameter of the formed part was φ1.47 mm, indicating that a bottomed microtube with a product diameter of φ1.71 mm and wall thickness of 120 µm can be fabricated.

**Figure 3:**
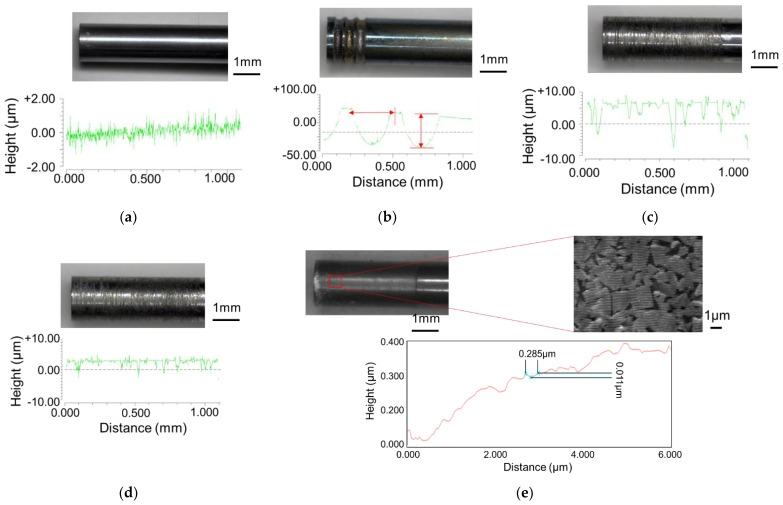
Textured punches: (**a**) mirror surface, (**b**) millimeter—textured (depth: 100 μm; approximately 2 grooves/mm), (**c**) 10 μm—textured (depth: 10 μm; ~10 grooves/mm), (**d**) 5 μm—textured (depth: 5 μm; approximately 10 grooves/mm), and (**e**) nanometer—textured (depth: 0.01 μm; approximately 3 grooves/μm).

Figure 3 shows the punches used in this study. Figure 3a shows a mirror-finished punch with a ground surface, and Figure 3b shows a millimeter-textured punch with a constant length of millimeter-sized grooves dug using an electric discharge machine on the mirror-finished punch (Figure 3a). Figure 3c shows a 10 µm-textured punch with 10 µm grooves in Figure 3a formed using abrasive paper with a grain size of 140. Figure 3d shows a 10 µm-textured punch with several micro-sized grooves of approximately 10 µm in depth and 100 µm in pitch on abrasive paper with a grain size of 400. The 5 µm-textured punches have several micro-sized grooves of approximately 5 µm in depth and 100 µm in pitch. Figure 3e shows a punch with several micro-sized grooves of approximately 100 µm in pitch and 5 µm in depth. The nanometer-textured punch shown in Figure 3e was produced by applying a nanoscale periodic groove structure (Ra = 0.099 µm) to the ground mirror punch shown in Figure 3a using ultrashort pulsed laser machining (Lips Works Co., Ltd., Ota-ku, Tokyo, Japan). The wavelength, pulse durations, average power output, and maximum frequency are 515 mm, 180–190 fs, 8.2 W, and 600 KHz, respectively. Figure 3e shows that the grooves are 0.01 μm deep with a pitch of 0.3 μm and are applied up to 3 mm from the punch tip, as observed using a scanning microscope at 10,000×. The nanogrooves are oriented parallel to the direction of the punch.

The test billets were cut from an A6063 aluminum alloy φ1.70 mm round wire and finished to a length of 4.0 mm. Table 1 lists the shape dimensions, grain size, mechanical properties, and microstructure of the billet [21]. The average grain size of the billets was 23.3 μm. The relationship between the true stress *σ* and true strain *ε* of the billet can be expressed by the hardening equation (Equation (1)), which is the power of the plasticity factor *F* [MPa] and the work-hardening index *n*. Because the mechanical properties are significantly affected by the microstructure, the mechanical properties should be evaluated based on the dimensions. The true stress σ and true strain ε were obtained from microcompression tests using billets 1.70 mm in diameter and 2.5 mm in length [18,19]. The compression ratio was 80%, and the compression rate was 0.1 mm/s. The plasticity factor, *F*, and work-hardening index, *n*, were derived from this microcompression test [18,19,21].

*σ* = *F ε^n^* (1)

Extrusion conditions were set at 0.1 mm/s ram speed and 1.5 mm ram stroke at room temperature, based on previous studies [21]. In this experiment, the extrusion test was repeated four times for each billet to ensure reproducibility. Scanning electron microscopy coupled with electron probe microanalysis (SEM-EPMA, JEOL JXA-8230, Akishima, Tokyo, Japan) was used to observe the surface properties and adhesion of the punch. The acceleration voltage for EPMA was 15 kV, and the measurement range was 2.5 mm (vertical) × 1.8 mm (horizontal). Electron backscatter diffraction (EBSD, JEOL JSM-6700F, Akishima, Tokyo, Japan) patterns were obtained to analyze the microstructure of the product. Grain orientation distribution and grain size were analyzed using EBSD to measure the effect of using a tool with reduced friction by a grooved punch on the material. A measurement of 800 μm × 0.15 mm was obtained from the tip of the extrusion. The specimens were mirror-polished with an abrasive, and the surface static stress was removed by ion milling. The measurement conditions included an acceleration voltage of 20 kV, irradiation current of 13 nA, working distance of 15 mm, and magnification of 500×. Step sizes of 0.5 and 0.1 µm were used for measurements at the extrusion tip and local measurements, respectively.

## 3. Experimental Results and Discussion

### 3.1. Extrusion Force–Ram Stroke Diagram and Metal Flow during Backward Microextrusion

**Figure 4:**
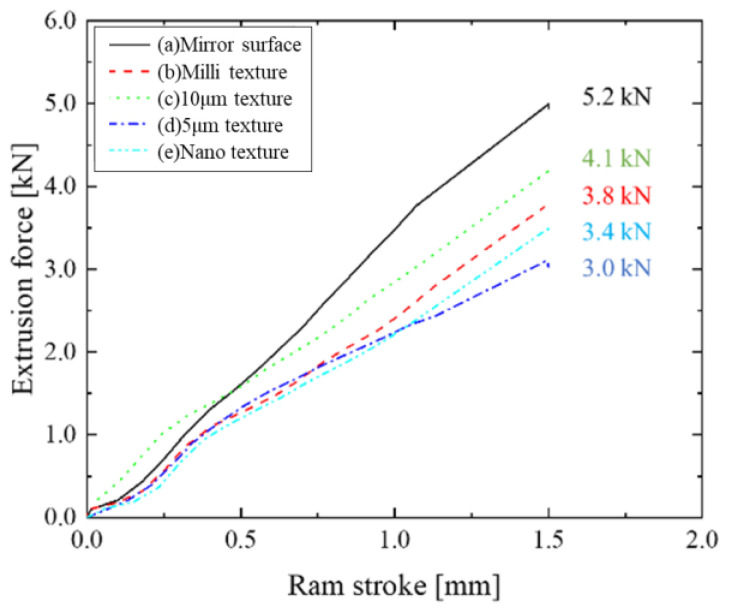
Extrusion force–ram stroke curve in each punch.

Figure 4 shows the extrusion force–stroke diagrams for the mirror punch, millimeter-textured punch, 10 µm-groove texture punch, 5 µm-groove texture punch, and punches with different surface properties of the nanotexture. The maximum extrusion load is 5.2 kN for the (a) mirror surface punch, which is the highest, compared to 3.8 kN for the (b) millimeter-textured punch; the force is reduced by the addition of texture. Based on the comparison of the micrometer-scale forces, that is, (c) 4.1 kN for the 10 µm texture and (d) 3.0 kN for the 5 µm texture, the force is reduced by decreasing texture size. 

**Figure 5:**
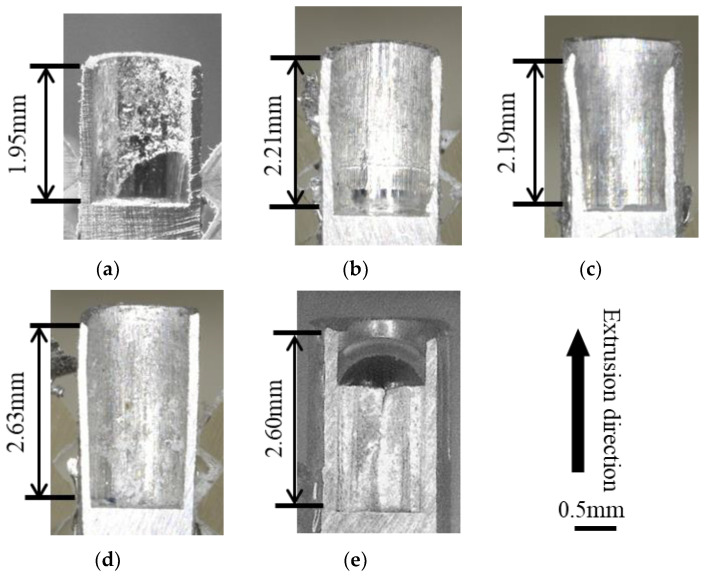
Longitudinal section cross-sectional images of the extrusion: (**a**) mirror surface, (**b**) millimeter-textured, (**c**) 10 μm-textured, (**d**) 5 μm-textured, and (**e**) nanometer-textured punches.

Figure 5 shows the cross-sectional shape of each punch after micro backward extrusion and the backward extrusion length (lb) for each product at a ram stroke of 1.5 mm. The lb values are 1.95, 2.21, 2.19, 2.64, and 2.60 mm for (a) mirror, (b) millimeter-textured, (c) 10 µm-textured, (d) 5 µm-textured, and (e) nanotextured punches, respectively. The lb is similar in length; however, the reduction in the textured true contact area possibly reduces friction and increases lb by facilitating appropriate material flow. The shorter backward extrusion length of the millimeter-textured and 10 µm-textured punches than that of the 5 µm-textured punch may be explained using millimeter-sized grooves in microscale machining generating two flows: one in the backward extrusion direction and another that enters the tool groove. By reducing the texture depth, the Al adhesion of the punch was broken, and friction was reduced, thereby resulting in smoother plastic flow and a longer backward extrusion length. Therefore, a friction reduction effect can be obtained by reducing the texture depth in the microscale plastic forming.

### 3.2. Evaluation of Adhesion to Punch

**Figure 6:**
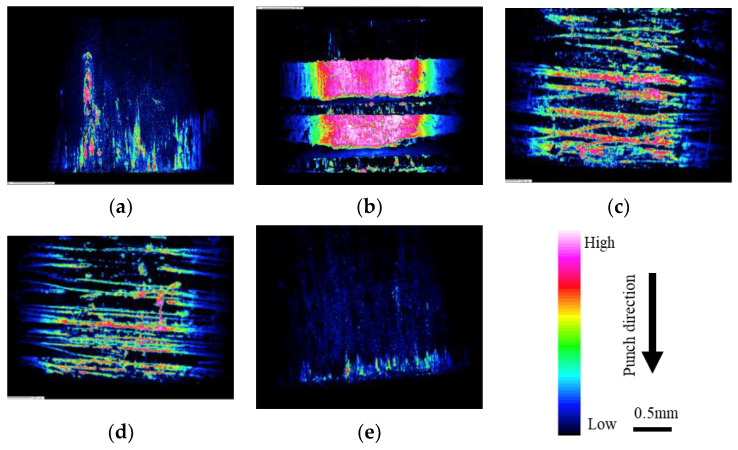
Evaluation of adhesion to punch via EPMA: (**a**) mirror surface; (**b**) millimeter-textured; (**c**) 10 μm-textured; (**d**) 5 μm-textured; and (**e**) nanometer-textured punches.

Figure 6 shows the amount of adhesion on the punch surface after processing with punches with different surface properties: (a) mirror punch, (b) millimeter-textured punch, (c) 10 µm-textured punch, (d) 5 µm-textured punch, and (e) nanotextured punch. EPMA was used to analyze the punch surface.

The experimental results showed that the textured punch broke the adherence of Al in the grooves on the circumference. Additionally, when comparing the amount of deposition between the two punches with different surface texture sizes, the 5 µm-groove punch shows less deposition than the 10 µm-groove punch, possibly because the pocket into which the material flows shrinks when the groove size is smaller; therefore, the adhesion can be broken into smaller pieces [21]. Moreover, the force of friction is reduced by breaking up the adhesion, resulting in a reduction in force. In particular, nanotextured punching causes the least amount of adhesion, which is considered to be due to the fine fragmentation of adhesion at the nanoscale.

### 3.3. Microstructure Analysis of the Extrusion

**Figure 7:**
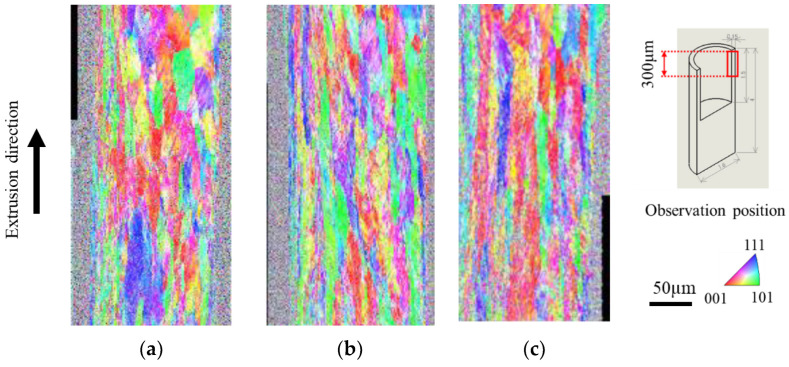
IPF map of the extrusion obtained using EBSD: (**a**) mirror surface, (**b**) 5 μm-textured, and (**c**) nanometer-textured punches.

**Figure 8:**
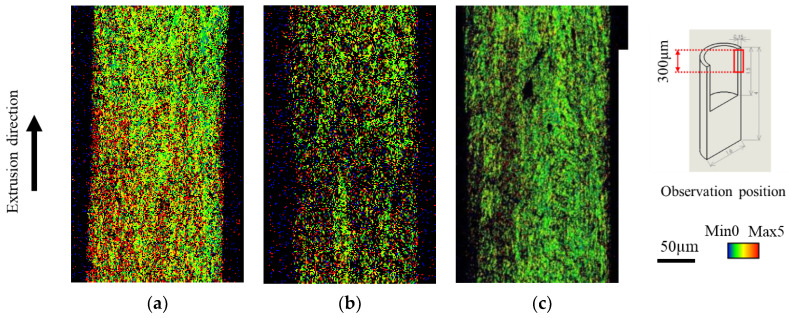
KAM map of the extrusion via EBSD: (**a**) mirror surface, (**b**) 5 μm-textured, and (**c**) nanometer-textured punches.

Figure 7 shows the inverse pole figure (IPF) map results obtained using mirror, 5 µm-textured, and nanotextured punches. The IPF map can be used to determine crystal orientation, which is defined by the crystal plane, by color. Grains at the leading edge of the extrudate flowed out without shearing. The material was sheared longitudinally, and the grain size increased toward the rear end of the material [21]. The measurement position was enlarged from 300 to 600 µm from the tip to exclude the non-steady state during the early stage of extrusion. Compared with the mirror punch, the texture punch was sheared, and the crystal grains were elongated vertically. The microtextured punches were sheared more strongly than the nanotextured punches, resulting in a larger vertical elongation. Therefore, the extrudate length was longer for the microtextured punch. Figure 8 shows the results of the kernel average misorientation (KAM) map, which is a quantitative method for evaluating the residual strain inside a sample based on the crystal orientation difference information. (a) In the specular punch, green and yellow colors with an azimuthal difference of 1–2° are mostly distributed at the tip of the material, whereas red and yellow colors with an azimuthal difference of 3–5° are mostly distributed at the rear end of the material. By contrast, (b) the 5 µm-textured and (c) nanotextured punch have a uniform strain exceeding 3–5°. This outcome suggests that the texture punches have low friction, which facilitates material flow; therefore, strain is uniformly introduced to accelerate the processing progress. The (a) specular punch has high friction, which hinders material flow, and the strain is considered to accumulate unevenly. Compared to the (b) 5 µm-textured punch, the (c) nanotextured punch shows negligible red distribution with an orientation difference of 5° or higher; additionally, the green areas, which have a smaller strain, are uniformly distributed. Grain deformation was homogenized by nanotexturing, and the grain size was refined without the accumulation of local plastic strain. The machining limit is expected to improve because a low-friction machining environment can be realized.

### 3.4. Comparison of Microtexture and Nanotexture Punches

**Figure 9:**
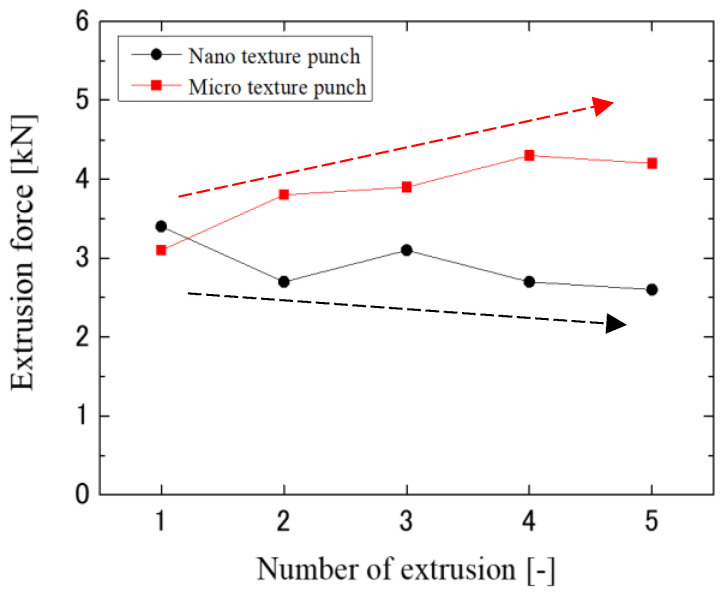
Extrusion force vs. number of extrusions.

**Figure 10:**
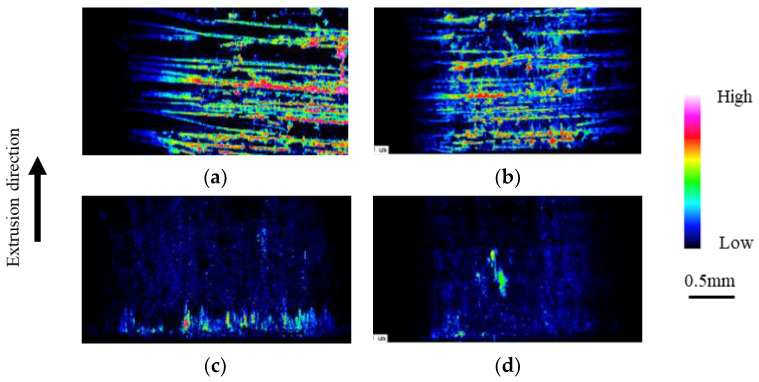
Evaluation of adhesion to punch via EPMA for 5 μm-textured punch: (**a**) 1st and (**b**) 5th extrusion; nanometer-textured punch: (**c**) 1st and (**d**) 5th extrusion.

**Figure 11:**
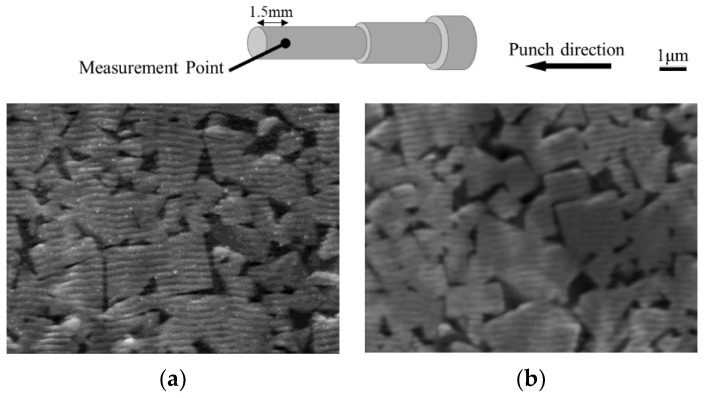
SEM images of the nanotextured punch before and after extrusion (punch side): (**a**) as received and (**b**) after extrusion (5th extrusion).

To investigate the anti-adhesion and anti-wear properties of micro- and nanotextured punches, a comparison in terms of the first and fifth extrusion cycles was conducted as shown in Figure 9. The nanotexture punch continues to reduce the force, whereas the 5 µm-textured punch tends to increase the extrusion force. Figure 10 shows the results of EPMA measurements of Al element adhesion on the punch surfaces after the first and fifth extrusion cycles. In the case of the 5 µm-textured punch, the EPMA results show increased adhesion in the grooves, and Al adhesion in the recesses of the grooves, which is not observed in the first cycle, is also observed in the fifth cycle. In the case of the 5 µm-textured punch, the grooves are worn out by the fifth cycle of processing, and the increased amount of adhesion is considered to have caused the increased extrusion force. However, the nanotextured punch shows no significant change in the amount of adhesion compared to that in the first punching, indicating that the effect of reducing the amount of adhesion is sustained. This finding suggests that the forces of adhesion and detachment are repeatedly applied to the textured part of the 5 µm texture, leading to the wear of the texture and, consequently, an increase in force because the friction reduction effect cannot be maintained. Figure 11 shows 10,000× SEM images of the nanotextured punch before and after extrusion. The observation of the side surface of the nanotextured punch shows that the texture is not worn away and retains its shape. The 5 µm-textured punch was textured perpendicular to the direction of punch travel, whereas the nanotexture punch was textured parallel to the direction of punch travel. The microtextured punches applied a force perpendicular to the edges of the texture ring, and the nanotextured punches applied a force parallel to the direction of the punch, which may have further damaged the texture ring.

Considering the lifetime of the texture and durability of the force reduction effect, the nanotextured punch is a tool with superior wear resistance and a longer life surface function than that of the microtextured punch. However, reducing the texture size to the nano-level and lower is limited by laser texturing technology, and advanced microsurface creation technology, such as ion beam texturing, is needed.

## 4. Conclusions

1. The tool surfaces were textured from the millimeter to nanometer scale using electrical discharge machining, polishing, and an ultrashort pulsed laser.
2. The extrusion force–stroke diagram for the micro-anteroposterior extrusion process increased the extrusion force gradually with an increase in stroke. The extrusion force was reduced by adding microscale texture to the punch surface.
3. The EPMA evaluation of the punch surface adhesion revealed that the punches with no texture and millimeter-scale texture showed more adhesion to the punch, and the amount of adhesion decreased as the texture size reduced.
4. IPF and KAM maps obtained via EBSD show that micro- and nano-textures on the punch surface improved material flow. A more uniform strain on the product was observed, particularly in the case of nano-textures.
5. Repeated experiments showed that the extrusion force and adhesion to the punch increased with increasing extrusion frequency for the microscale texture. For the nanotextured punches, the extrusion force decreased with increasing extrusion frequency, while adhesion to the punches decreased.

Future research will include an investigation into texture direction in nanotextured punches and their application to the preparation of biomaterials based on magnesium and titanium.

## Figures and Tables

**Table 1:** Dimension and properties of the AA6063 billet [21].

Item	Value and Figure
Shape of billet	φ 1.70 × 4 (mm)
Vickers hardness	33.2 (HV)
*F*	169.0 (MPa)
*n*	0.29
Microstructure	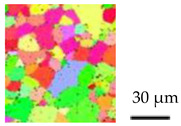
Grain distribution	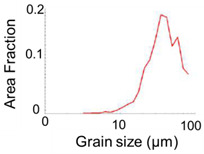
Average grain size	23.3 (μm)

## Data Availability

Not applicable.
